# The Impact of Ultraviolet-B Radiation on the Sugar Contents and Protective Enzymes in *Acyrthosiphon pisum*

**DOI:** 10.3390/insects12121053

**Published:** 2021-11-25

**Authors:** Chunchun Li, Weining Yuan, Yuping Gou, Kexin Zhang, Qiangyan Zhang, Jing-Jiang Zhou, Changzhong Liu

**Affiliations:** 1 Biocontrol Engineering Laboratory of Crop Diseases and Pests of Gansu Province, College of Plant Protection, Gansu Agricultural University, Lanzhou 730070, China; lichch1992@163.com (C.L.); nanjueyuan@126.com (W.Y.); gouyp1988@163.com (Y.G.); zhangkx199404@163.com (K.Z.); zhangqiangyan2@163.com (Q.Z.); jjzhou@gsau.edu.cn (J.-J.Z.); 2 State Key Laboratory of Green Pesticide and Agricultural Bioengineering, Ministry of Education, Guizhou University, Huaxi District, Guiyang 550025, China

**Keywords:** *Acyrthosiphon pisum*, UV-B radiation, protein, glycogen, trehalose, enzyme activity

## Abstract

**Simple Summary:**

The decrease of stratospheric ozone contributes to a significant increase in solar ultraviolet (UV-B) radiation. This effect has led to investigation on the impact of increased UV-B radiation on insect physiology. Pea aphid is a worldwide important agricultural pest and is difficult to control due to its small size, high fecundity, and non-sexual reproduction. As such, there is a need for study of the effects of UV-B radiation on aphid physiology, to raise awareness of the mechanisms of aphid responses to UV-B stress. The results reported here revealed that UV-B radiation can lead to an increase in sugar contents in both red and green morphs of this aphid species, and confirmed the effects of UV-B radiation on aphid physiology by means of influencing protective enzyme activity.

**Abstract:**

Natural and anthropogenic changes have been altering many environmental factors. These include the amount of solar radiation reaching the Earth’s surface. However, the effects of solar radiation on insect physiology have received little attention. As a pest for agriculture and horticulture, aphids are one of the most difficult pest groups to control due to their small size, high fecundity, and non-sexual reproduction. Study of the effects of UV-B radiation on aphid physiology may provide alternative control strategies in pest management. In this study, we examined the effects of UV-B radiation on protein and sugar contents, as well as the activities of protective enzymes, of the red and green morphs of the pea aphid over eight generations. The results indicated a significant interaction between UV-B radiation and aphid generations. Exposure of the pea aphids to UV-B radiation caused a significant decrease in the protein content and a significant increase in the glycogen and trehalose contents at each generation as measured in whole aphid bioassays. The enzyme activity of superoxide dismutase (SOD), peroxidase (POD), and catalase (CAT) of the pea aphids changed significantly at each generation with UV-B treatments. The SOD activity increased over eight generations to the highest level at G_7_ generation. However, the enzyme activity of CAT first increased and then decreased with UV-B treatments, and POD mostly gradually decreased over the eight generations. Therefore, UV-B radiation is an environmental factor that could result in physiological changes of the pea aphid. Moreover, our study discovered that red and green aphids did not display a significant consistent difference in the response to the UV-B treatments. These results may prove useful in future studies especially for assessing their significance in the adaptation and management against UV-B radiation.

## 1. Introduction

The decrease of stratospheric ozone makes solar ultraviolet (UV-B) radiation increase significantly [1], and has great and sometimes adverse effects on the carbon and nitrogen cycle of the whole biosphere [2], living habitat [3], biological characteristics [4], physiology, and biochemistry [5], and this is likely to affect aphids. 

In recent years, the destruction of the stratospheric ozone layer in the Earth’s atmosphere by the emission of anthropogenically generated pollutants [6,7] has greatly increased interest in the effects of solar ultraviolet radiation, especially ultraviolet-B (UV-B) radiation, which is considerably more harmful to living organisms than UV-A because of its shorter wavelength and consequently higher energy levels [8]. UV-B has a wide range of effects on the growth, biochemistry, physiology, and population structure of organisms. There is a consensus that gradual UV-B radiation intensity can result in serious climate change, including increase in global temperature, imbalances in natural ecosystems, and risks for the survival of living organisms [7]. An increasing body of evidence suggests that UV irradiation is one of the most ubiquitous environmental hazards affecting every organism [9]. It is considered to be an environmental factor that induces oxidative damages to organisms through the production of reactive oxygen species (ROS), and causes damage to DNA, membrane lipids, and proteins [10,11,12,13,14]. UV-B radiation has been reported to affect the metabolic rates of both *Aedes albopictus* and *Culex pipiens*, whose survival rate were also significantly lower in full-sun compared to shade and no UV-B conditions [5]. However, information about oxidative stress induced by UV irradiation in insects is scarce, including for aphids, and relatively few studies have examined the responses of protective enzymes in insects under UV irradiation. 

The protective enzymes such as superoxide dismutase (SOD), peroxidase (POD), and catalase (CAT) are an important component of innate insect immunity. The main functions of these enzymes are to clear the free radicals from the body, and protect insects against the damage caused by reactive oxygen species (ROS) or other peroxide free radicals [15]. In addition, the activity of protective enzymes is often used as a measure of insect physiological state [16]. Under normal physiological conditions, these enzymes, and free radicals are maintained in a state of equilibrium in insects. However, when insects are exposed to UV-B [17,18], heat stress [19], insecticides [20,21], pathogens [22,23], the levels of the enzymes and free radical changes, and the protective enzymes are activated to decompose and metabolize free radicals, to maintain normal physiological activities in the body [24]. It has been reported that UV light irradiation induced superoxide radical and intensified the activity of protein oxidation processes in *Helicoverpa armigera* adults [17], and raised the activities of protective enzymes in its body to resist the damage of the radicals in the aphid *Sitobion avenae* [25]. 

The pea aphid, *Acyrthosiphon pisum* (Harris), is a soft-bodied hemimetabolous insect that is distributed globally. Furthermore, *A. pisum* has red and green body colour polymorphisms. The green pea aphid is widely distributed all over the world and has been reported since 1776 [26], while the red pea aphid was first reported by Harrington in 1945 [27]. Laboratory crosses between red and green morphs indicates that the body colour assay of pea aphids is genetically determined, and its morphs remain distinct between generations because the aphids reproduce parthenogenetically [28,29]. However, some environmental factors (e.g., symbiotic microorganisms) are also considered to affect aphid body colour [30]. 

*A. pisum* lives year-round under direct sunlight and is not capable of escaping from UV-B [5,31]. The aphids voraciously suck fluid from plants and secret honeydew, causing the leaves of plants to mould, wither, and even die, therefore reducing crop yields [32]. They can also transmit 25 types of viruses by sap-feeding, causing enormous production losses of cash crops [33]. In recent years, because of the influence of many factors (e.g., UV radiation), the pea aphid population has become one of the dominant pests in alfalfa (*Medicago sativa*), and the importance of the red pea aphid has gradually increased in China [34]. This has enhanced the research on the pea aphids [35,36,37,38], and on the effects of UV-B treatment on biological characteristics of aphids. Yuan et al., [39,40] showed that ultraviolet radiation inhibited the growth and reproduction of pea aphids, and the effect of this inhibition was proportional to the radiation intensity and time. Moreover, with prolonged exposure to UV-B radiation over time, the average body length of red pea aphids was shortened, and the average weight reduced [40]. 

In the current study, we aimed to elucidate the effect of UV-B treatment on the sugar contents and protective enzyme activity in the red and green morphs of the pea aphid over eight generations. We compared the effect of UV-B treatments on the contents of protein, glycogen and trehalose, and the activity of SOD, POD, and CAT in the red and green morphs. This study helps to inform approaches for further study of the defense mechanism of the pea aphids to environmental changes and support the development of new approaches for pest management.

## 2. Materials and Methods 

### 2.1. Insect Culture

The initial population of the pea aphids *Acyrthosiphon pisum* Harris used in this study was established from a single parthenogenetic female collected from an alfalfa field in Gansu Province, China, and maintained in Biocontrol Engineering Laboratory of Crop Diseases and Pests. The pea aphids were reared on the fava bean plant (*Vicia faba* L.) leaves in the laboratory for eight generations. Briefly, leaves of fava bean were placed on a Petri dish (9 cm in diameter) with a piece of moist absorbent cotton around the petioles and the back side of the leaf facing up. The red and green aphids sourced from these leaves were then separately introduced into the additional Petri dishes with bean leaves and reared separately in two incubators at 25 ± 1 °C, 70% relative humidity and a photoperiod of 16L: 8D. Adult pea aphids that molted within 12 h from 4th instar aphids were used for tests.

### 2.2. Mode of UV-B Treatment

A UV-B lamp (Hebei Zhonglian Qitong Information Technology Co., Ltd., ZW40D17W, Hebei, China) was used as the source of artificial UV-B radiation. The nymphs (<12 h) produced by the adult aphids were fed on bean leaves for 12 h and then treated at 4 W/m^2^ intensity of UV-B radiation for 20 min, 30 min, 40 min, and 50 min. The distance between the aphids and the UV-B lamp was 30 cm. The aphids treated under an incandescent lamp at 4 W/m^2^ intensity (New Light Source Lighting Technology Co., Ltd., ST58, Haining, China) were served as control. The UV-B treatment and the control treatment were performed with 20 either red or green aphid morphs in a Petri dish with leaves for each exposure duration. The aphids were irradiated once a day for the specific exposure duration until the adult stage (about 6 days). All treatments were repeated 3 times. 

After 6-day exposure, five red aphids and five green adult aphids were collected as G_0_ treated aphids from each dish and stored at −80 °C for the following experiments. Then the newly emerged (<12 h) nymphs from the remaining adult aphids were transferred to new Petri dishes with fresh leaves and used for next run of the UV-B treatments. This test procedure was repeated 8 times, and produced aphids were used to the treatment for 8-generation (G_0_–G_7_). A total of 2400 nymphs (20 nymphs × 5 treatments × 3 replicates × 8 generations) was used in this experiment.

### 2.3. Determination of Trehalose and Glycogen Content

The measurements of trehalose and glycogen contents in the red and green aphid morphs were determined mainly following the method used by Handel & Day [41]. Five adult aphids from each generation stored at −80 °C were rinsed with sterile distilled water and placed on filter paper to absorb the moisture on the surface of the aphids. After the aphids were weighed, they were put together in a 1.5 mL centrifuge tube containing 50 μL of 10% trichloroacetic acid. The aphids were then ground with a grinding stick in the centrifuge tube. A total of 350 μL of 10% trichloroacetic acid solution was used to wash the grinding stick twice and added into the centrifuge tube. The aphid homogenate was then centrifuged at 5000 rpm for 5 min at 20 °C. The supernatant was transferred to a new 1.5 mL centrifuge tube, and the precipitate was discarded. Then, 800 μL of absolute ethanol was added to the supernatant, mixed and incubated at 4 °C overnight (about 16–17 h). Next day, the mixture was centrifuged at 10,000 rpm for 20 min. The resulting supernatant was transferred to a 1.5 mL centrifuge tube and used as the trehalose assay solution. The white precipitate was dissolved in 1000 μL of sterile distilled water and transferred to another 1.5 mL centrifuge tube as glycogen assay solution. 

200 μL of the trehalose assay solution was mixed with 1000 μL of 0.15 N H_2_SO_4_ solution and heated in a boiling water bath for 10 min. After the mixture was cooled down, 1000 μL of 30% KOH was added. Then the mixture was boiled in a water bath for 10 min, and kept at room temperature for 20 min. The resulting solution (200 μL) was added to the microplate. The trehalose content and glycogen content were determined by measuring the absorbance value of the samples at 620 nm and 630 nm, respectively, with the spectrophotometer (Koromee Scientific Instruments Co., Ltd., UV-6100, Shanghai, China). The trehalose and glycogen contents were calculated from the absorbance value using D-glucose as standard. Each UV-B treatment was repeated three times.

### 2.4. Preparation of Enzyme Solution

The red and green adult aphids from each generation were collected as described above. The aphids (*n* = 5 for each colour morph) were weighed and together placed in a pre-cooled test tube, and then 1 mL of 50 mmol/L phosphate buffered saline (PBS, pH 7.8) was added to create a suspension. The suspension was ground using a glass homogenizer in an ice bath, homogenized by ultrasonic disruption for 10 min, and centrifuged at 10,000 rpm at 4 °C for 10 min. The supernatant was collected, stored at −80 °C and used as the enzyme solutions to measure the total amount of protein and the activity of protective enzymes. 

### 2.5. Protein Content Determination

Protein content in red and green aphids were determined according to Lowry et al. methods [42]. Seven different concentrations (0, 0.02, 0.04, 0.06, 0.08, 0.10, and 0.12 mg/mL) of the bovine serum albumin (BSA) (99.5%, Hubei Jiufenglong Chemical Co., Ltd., Wuhan, China), were used to construct the standard curve. About 50 μL of the enzyme solution was transferred into a well of 96-well microplate and addition of 200 μL Coomassie brilliant blue G-250 to each well. The protein concentration was quantified spectrophotometrically at 595 nm. The protein contents were calculated based on the standard curve of BSA. Each UV-B treatment for each morph and each generation was repeated three times.

### 2.6. Protective Enzyme Activities of Pea Aphids

The superoxide dismutase (SOD) activity was determined according to Beauchamp & Fridovich methods [43]. We mixed 125 μL of the reaction fluid with 5 μL of the enzyme solution and 50 μL of 13 mmol/L riboflavin. The 5 μL enzyme solution was replaced with 5 μL of 50 mM PBS in the control groups. After 15 min exposure to light at 4000 Lx and 25 °C, the solution was immediately protected from light and the absorbance at OD_560_ of the solution was determined immediately. The measurement was measured 3 times. The SOD enzyme activity was then measured as the OD_560_ value and calculated using the amount of enzyme required for 50% inhibition as an enzyme activity unit (U). Enzyme activity was expressed as the change in the OD_560_ value per unit of time and unit of protein. 

The catalase (CAT) activity was determined according to Chance & Maehly methods [44]. In short, 100 μL of H_2_O_2_ (8%) solution mixed with to 190 μL of 50 mM PBS (pH 7.8). Then, 10 μL of the enzyme solution was added to the mixture. The OD240 was measured immediately at 25 °C every 1 min for 3 min. In the control group, the PBS buffer was used instead of the enzyme solution. The experiment was repeated 3 times. The amount of enzyme that reduces OD240 by 0.1 unit in 1 min is expressed as 1 unit of CAT activity.

The peroxidase (POD) activity was determined according to Shan et al. [45]. The sample mixture contained 235 μL of 50 mM PBS buffer (pH 7.8), 20 μL guaiacol and 15 μL of the enzyme solution. Subsequently, 15 μL of 26 mM H_2_O_2_ was quickly added. The samples were placed at room temperature for 5 min, and the colorimetry was measured at a wavelength of 470 nm every 1 min for 10 min. The reactions were measured 3 times. The POD activity was expressed as an increase of 0.01 of A470 per minute. The control consisted of the PBS buffer instead of enzyme solution. 

### 2.7. Statistical Analysis

Data were expressed as a mean ± standard deviation (S.D.) for the 3 replicates for the 2 colour morphs, 8 generations, and 4 treatment times. All statistical analyses were performed using IBM SPSS Statistics version 23.0 (Chicago, IL, USA), with UV-B treatment, and generation included as factors. The general linear model (GML) procedure of the Statistical Analysis System (SAS) (https://www.sas.com/en_us/home.html; accessed on 29 July 2021) was used to analyze the means when a significant interaction between generations and UV-B treatments. One-way ANOVA was conducted to examine the effects of UV-B radiation treatment and generation on the test parameters (protective enzyme activity, sugar and protein contents) followed by Tukey’s honestly significant difference (HSD) test. In addition, these test parameters were compared between G_0_ and G_7_ on red and green morphs, respectively. Differences between the means were tested for significance at the 0.05 significance level. 

## 3. Results 

### 3.1. Interaction between UV-B Treatment and Generation 

There was a statistically significant interaction between the UV-B treatment and growth generation on the glycogen content, the protein content, the SOD activity, and the CAT activity of both aphid colour morphs (*p* < 0.001) (Table 1). The interaction of the two factors (generation and UV-B treatment) was not significant only in the green aphids for the trehalose content (F = 1.48, df = 28, *p* > 0.05) and in the red aphids for the POD activity (F = 0.54, df = 28, *p* > 0.05). Therefore, we used one-way ANOVA and Tukey’s HSD test to separately compare the effect of UV-B treatment at G_0_–G_7_ generation and the effect of growth generation under different UV-B treatment.

### 3.2. Effects of UV-B Treatments on the Trehalose Content of Aphids 

**Figure 1:**
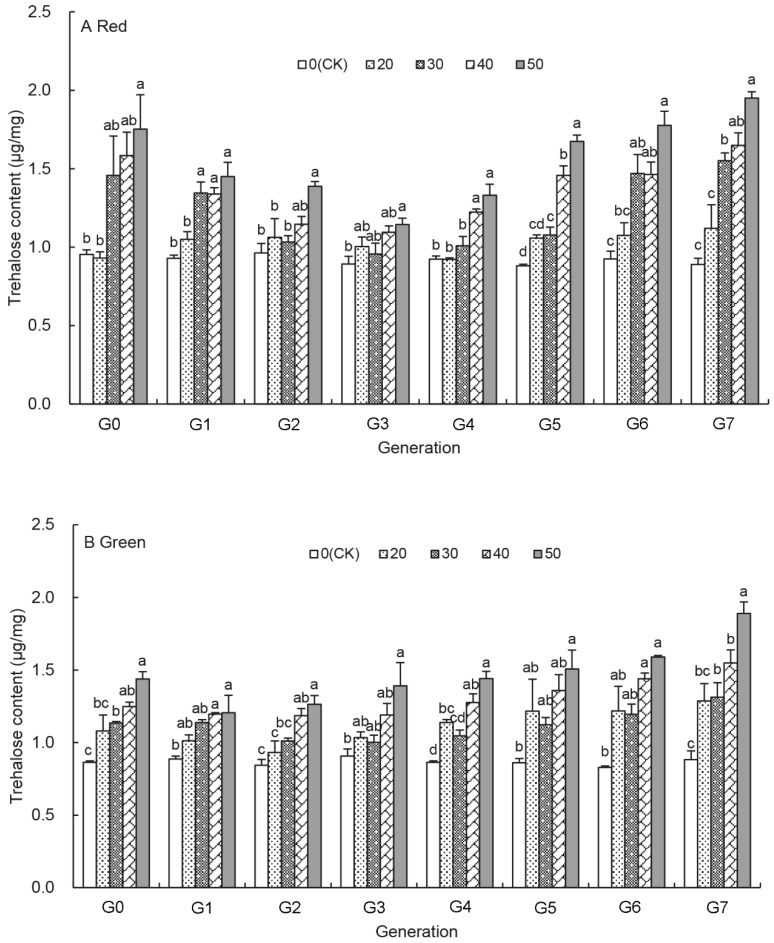
Effects of UV-B radiation on the trehalose content of *A. pisum* at each generation(G_0_–G_7_). Different lowercase letters between UV-B radiation are statistically different at a same generation in red (**A**) and green (**B**) *A. pisum* (*p* < 0.05, Tukey-HSD test).

Overall, the trehalose content increased with the duration of the UV-B treatments (20–50 min) for each generation (G_0_–G_7_), and it peaked with the 50 min UV-B treatment at G_7_ generation (Figure 1A,B). During earlier generations (G_0_–G_3_) the trehalose content of the UV-B treated red aphids dropped with increasing generation, the lowest trehalose content was seen at G_3_ generation. Then, the trehalose content began to increase with the generation under most UV-B treatments (Figure 1A and Figure S1A). At G_7_ generation, the trehalose content was significantly higher in the 30 min–50 min treated red aphids than in untreated control aphids and increased significantly from 1.55 ± 0.05 μg/mg in the 30 min treated aphids to 1.65 ± 0.08 μg/mg and 1.95 ± 0.04 μg/mg in the 40 min and 50 min treated aphids, respectively, 1.74–2.19 times higher than that of the control aphids (Figure 1A). Like in the red aphids, the trehalose content in the UV-B treated green aphids was also highest in the 50 min treated aphids for each generation. However, unlike the U-shape response in the treated red aphids, the shape of the trehalose content is indeterminate over generations in the treated green aphids (Figure 1B and Figure S1B). There was no significant difference in the trehalose contents between the 20 min UV-B treated and untreated control aphids, but in the 30 min–50 min UV-B treated aphids, the change of trehalose content was significant, and the content was higher compared to that of untreated aphids (Figure S1A,B).

### 3.3. Effects of UV-B Treatments on the Glycogen Content of Aphids

**Figure 2:**
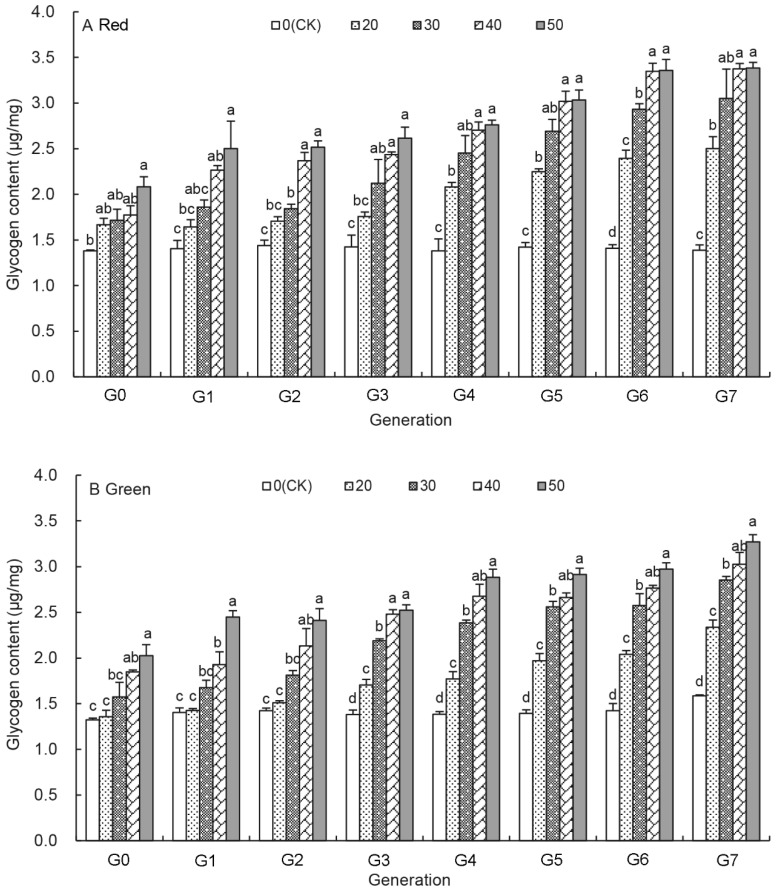
Effects of UV-B radiation on the glycogen content of *A. pisum* at each generation (G_0_–G_7_). Different lowercase letters between UV-B radiation are statistically different at a same generation in red (**A**) and green (**B**) *A. pisum* (*p* < 0.05, Tukey-HSD test).

For the red colour morph, Figure 2A reveals that the glycogen content increased as the UV-B treatment duration increased from 20 min to 50 min in each generation and was significantly higher than that of untreated aphids after G_4_ generation (Figure 2A). Moreover, the glycogen content in untreated aphid was relatively stable over generations, whereas it increased from G_0_ to G_7_ generation at each UV-B treatment in the UV-B treated aphids (Figure S2A). At G_7_ generation, the glycogen content had increased significantly by the UV-B treatment and ranged from 2.50 ± 0.13 μg/mg for the 20 min treatment to 3.39 ± 0.06 μg/mg for the 50 min treatment, which was 1.80–2.44 times higher than those of the untreated aphids (Figure 2A).

Figure 2B shows that the effect of UV-B treatments on the glycogen content in the green pea aphids. Like the red aphids after UV-B treatments, the glycogen content in the treated green aphids increased with the prolonged UV-B treatments at each generation and increased as the generation progressed at same UV-B treatment (Figure 2B and Figure S2B). The glycogen content at G_7_ generation was the highest under each UV-B treatment, which was 1.47–2.06 times higher that of the untreated control aphids (Figure 2B). 

### 3.4. Effects of UV-B Treatments on the Protein Content of Aphids 

**Figure 3:**
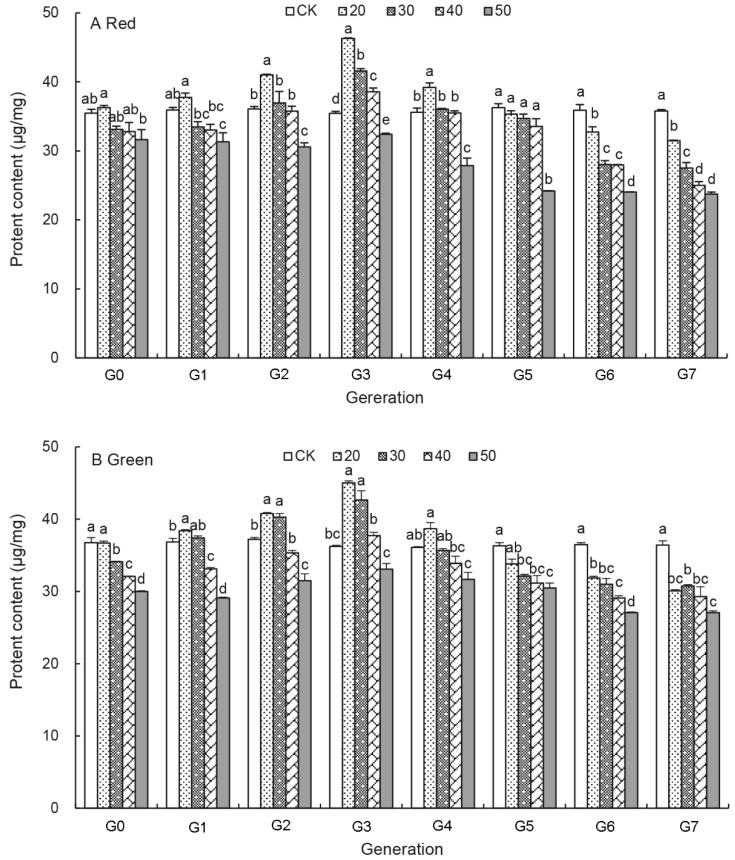
Effects of UV-B radiation on the protein content of *A. pisum* at each generation (G_0_–G_7_). Different lowercase letters between UV-B radiation are statistically different at a same generation in red (**A**) and green (**B**) *A. pisum* (*p* < 0.05, Tukey-HSD test).

The protein content of the red and green aphids before and after UV-B treatments is shown in Figure 3. It ranged from 23.75 ± 0.29 μg/mg to 46.28 ± 0.11 μg/mg in the red aphids, and 27.09 ± 0.24 μg/mg to 45.02 ± 0.28 μg/mg in the green aphids (Figure 3A,B). In both aphids, the protein content first increased, then decreased under the UV-B treatments in G_0_ to G_4_ generation (also Table S1). The highest protein content occurred in the 20 min treatment at G_3_ generation for both aphids. After G_3_ generation it gradually decreased with increasing generation with the lowest protein level recorded with UV-B treatment duration of the 50 min treatment at G_7_ generation. Compared with those of untreated aphids, the protein content was significantly reduced by 8.77–33.69% in the treated red aphids, and 12.56–25.70% in the treated green aphids at G_6_ and G_7_ generation. However, without the UV-B treatments (control), the protein content remained stable over generations in both aphids.

### 3.5. Effects of UV-B Treatment on SOD Activity of Aphids 

**Figure 4:**
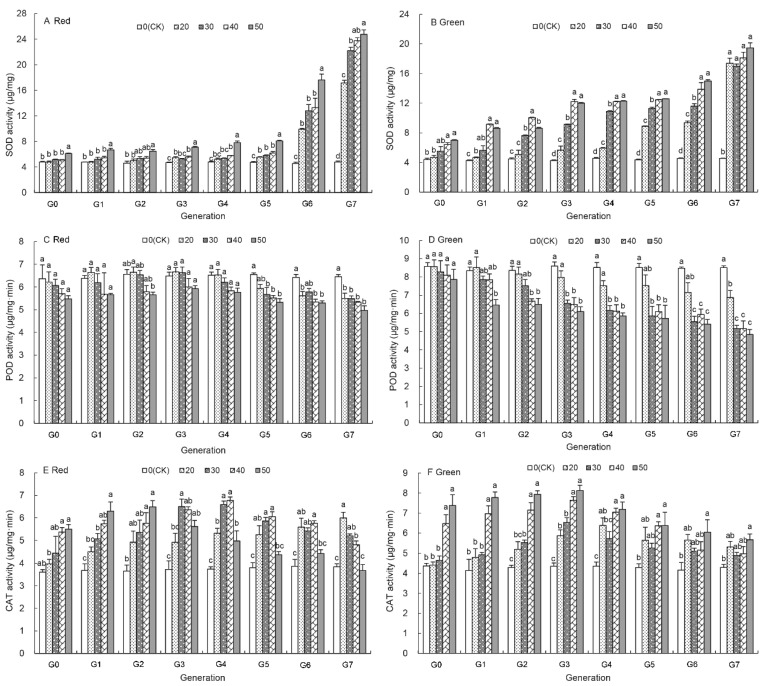
Effects of UV-B radiation on the protect enzyme activity of *A. pisum* at each generation (G_0_–G_7_). (**A**,**B**): Superoxide dismutase (SOD); (**C**,**D**): Peroxidase (POD); (**E**,**F**): Catalase (CAT). Different lowercase letters between UV-B radiation are statistically different at a same generation in red (**A**,**C**,**E**) and green (**B**,**D**,**F**) *A. pisum* (*p* < 0.05, Tukey-HSD test).

Figure 4A shows the effect of UV-B treatments on the SOD activity of the red aphids. The SOD activity at G_0_, G_1_ and G_2_ generation was not significantly affected by the 20–40 min UV-B treatments and was significantly higher after G_5_ generation compared to those of the control aphids. It was significantly increased by the 50 min UV-B treatment compared to that of the control aphids at each generation (Figure 4A). The SOD activity was highest at G_7_ generation over the generations and increased as the UV-B treatment duration increased from 17.16 ± 0.41 μg/mg by the 20 min treatment to 24.75 ± 0.69 μg/mg by the 50 min treatment, which are 3.61 and 5.20 times higher than those of the control aphids (Figure 4A). 

Similarly, the SOD activity at each generation of the green aphids increased with the UV-B treatment duration and over generations (Figure 4B and Figure S3B). At the G_7_ generation it increased by 2.73–3.28% from that of the control aphids to 17.39 ± 0.69 μg/mg, 16.98 ± 0.31 μg/mg, 18.11 ± 0.77 μg/mg and 19.47 ± 0.68 μg/mg under the UV-B treatments for 20 min, 30 min, 40 min, and 50 min, respectively. 

### 3.6. Effects of UV-B Treatment on the POD Activity of Aphids 

Unlike the activity of other enzymes, the POD activity was reduced by the UV-B treatments (Figure 4C,D). It was significantly lower than that of untreated aphids after G_5_ generation under the 30 min and 50 min treatments in the red aphids (Figure 4C) and in the green aphids (Figure 4D). At G_7_ generation, the POD activity of the red and green aphids was reduced by 22.93% and 43.07% than that of the control aphids by 50 min UV-B treatment, respectively. Moreover, the POD activity of the red aphids was not significantly changed by the 20–50 min UV-B treatments even at G_7_ generation, whereas the POD activity of the green aphids was significantly decreased from 8.60 ± 0.23 μg/mg·min to 4.85 ± 0.27 μg/mg·min after G_3_ generation by the UV-B treatments (Figure 4D and Figure S3C,D).

### 3.7. Effects of UV-B Treatment on the CAT Activity of Aphids

The CAT activity in both untreated red and green aphids remained unchanged over the generations (Figure 4E,F). However, the UV-B treatments resulted in an increase of the CAT activity between G_0_ to G_2_ generations in both colour morphs in a time-dependent fashion with the highest activity in the treated aphids treated for 50 min (Figure 4E,F). After G_3_ generation of the red aphids and G_4_ generation of the green aphids, the CAT activity decreased under the UV-B treatments. However, it was not reduced below the level of the untreated aphids at G_3_–G_6_ generation. At G_6_ and G_7_ generation of the green aphids, the CAT activity was still on average higher by 32.01%, and 21.31% in the 20 min to 50 min treated aphids than that of the control, respectively (Figure 4F). Only by the 50 min treatment at G_7_ generation was the CAT activity of the red aphid reduced below that of the untreated aphids, but they did not differ significantly (Figure 4E). 

### 3.8. Comparison of UV-B Treatments on Sugar and the Enzyme Activity at G_0_ and G_7_ Generations

**Figure 5:**
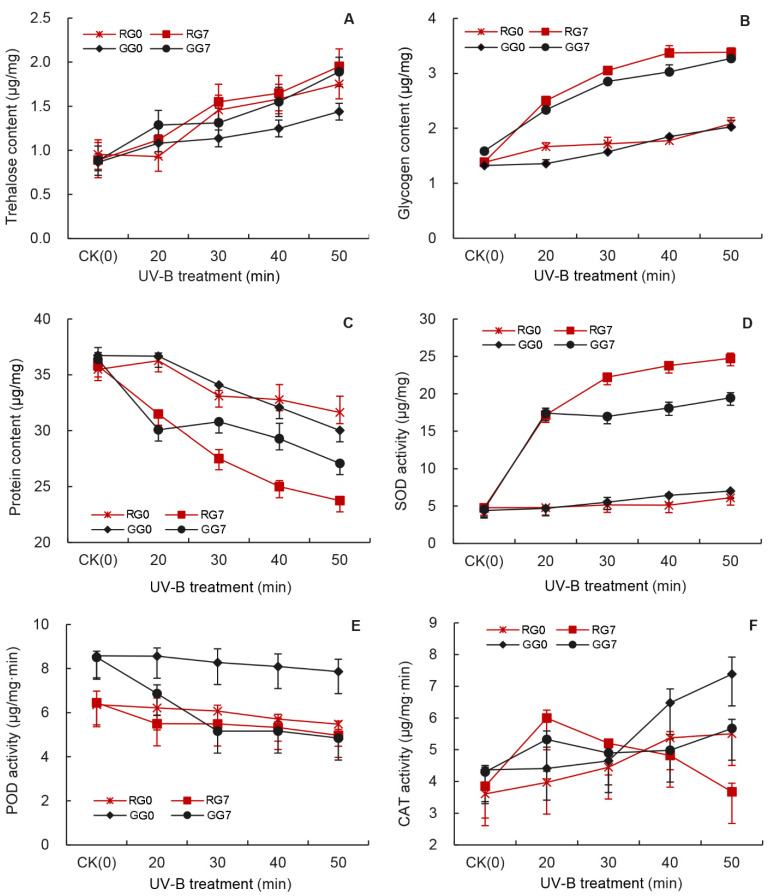
Comparison of UV-B treatment on sugar contents and the activity of protective enzymes of red (R) and green (G) pea aphids at G_0_ and G_7_ generations. (**A**): Trehalose content; (**B**): Glycogen content; (**C**): Protein content; (**D**): Superoxide dismutase (SOD); (**E**): Peroxidase (POD); (**F**): Catalase (CAT).

Figure 5 summarizes the effects of the UV-B treatments on the contents of total protein, glycogen and trehalose, and the activity of CAT, SOD, and POD at G_0_ and G_7_ generations. There was no significant difference in all measured parameters in the untreated aphids at G_0_ and G_7_ generations (Figure 5). The UV-B treatments caused an increase in the trehalose content, the glycogen content, the SOD activity, and the CAT activity, and a decrease in the protein content and the POD activity. The 20 min UV-B treatment caused a significant change in the glycogen content, total protein content, and the SOD activity between G_0_ and G_7_ generations (Figure 5B–D). However, the trehalose content, the POD activity, and the CAT activity between G_0_ and G_7_ generations were not changed significantly by the 20 min treatment (Figure 5A,E,F). In fact, the trehalose content between the generations was not significantly affected even under the 50 min treatment for both red and green aphids (Figure 5A). As the UV-B treatment duration increased, the total protein content and the POD activity decreased, while the glycogen content and the SOD activity significantly increased (Figure 5B,D).

At G_7_ generation, the protein content of the red aphid was lower than that of the green aphid under the 30 min to 50 min UV-B treatments, suggesting that the UV-B treatments may have a relatively greater impact on the protein content of the red pea aphids (Figure 5C). On the other hand, the UV-B treatments had a greater effect on the POD activity in the green aphids than in the red aphids (Figure 5E).

## 4. Discussion

The current study reports the long-term effects and possible mechanisms of UV-B on the protective enzyme activity and nutritional dynamics of the red and green morphs of pea aphids over eight generations.

Energy storage in arthropods has significant implications for survival and reproduction [46]. The primary energy sources are carbohydrates and proteins [47,48]. This study reveals a significant increase in the glycogen content after the UV-B treatments over eight generations (Figure 2) consistent with the response of insects to low temperature treatment [49,50]. The increase in glycogen content could be an adaptation mechanism of insects to adversity. In other research on *A. pisum*, the elevated CO_2_ levels also initiated aphid emergency responses by increasing glucose metabolism [51]. A similar trend was observed in the trehalose content of UV-B treated aphids (Figure 1), participating in the body’s energy supply as an energy substance as in diapausing pupae of the silkmoth, *Philosamia cynthia* [52]. Trehalose is a non-reducing sugar and the principal sugar circulating in the haemolymph of most insects. It also protects insects from the deleterious effects of osmotic, anoxic and other environmental treatments [53,54,55]. These results suggest that UV-B radiation is a negative environmental factor for *A. pisum*.

Our results revealed that the protein contents in the UV-B treated aphids were significantly lower than in control aphids between G_0_ and G_3_ generation, which is consistent with Ahsaei et al., who showed that the reproduction ability of the pea aphid was closely correlated with the protein contents in its body [56]. It is possible that more immune response-related proteins and enzymes were produced under the UV-B radiation. The protein content of insects is for development, maintenance, morphogenesis and reproduction, and cell osmotic pressure [57,58]. Based on previous research, protein supplements enhanced *Bactrocera minax* survival, mating, and fecundity [59]. More interesting, proteins may even be of more importance for aphids as there are three generations present within one viviparous female [60]. As the aphids grew, the long-term UV-B radiation resulted in the inhibition of protein synthesis and protein transportation, the protein content continuously declined from G_4_ to G_7_ generation (Figure 3 and Table S1). These results confirmed that a long period of UV-B treatment led to inhibitory effects on the development and population growth of the pea aphid [39,40].

The green aphid morphs accumulated higher protein than the red aphid morphs under the long-term UV-B radiation (Figure 3), which is in consistent with previous research that the green aphids produced more offspring than the red aphids [61] and that the green morph is considered to be batter adapted to cold conditions than red aphids, because they occur earlier in the vegetative season [62]. However, red aphids are more abundant than the green aphids during warmer months [63,64]. The red *A. pisum* aphids were more sensitive and showed a higher increase in the glycogen contents under the UV-B treatments (Figure 5B). They accumulated significantly more glycogen and trehalose than the green aphids as reported previously [61]. Proteins account for 42.14% of total energy reserves in the green aphids and 22.06% in the red aphids [56]. The mechanism for these physiological differences between the red and green aphids is not clear. An increase of energy reserve contents in *A. pisum* body led to their increased mobility [56]. Interestingly, winged aphids have a lower reproductive potential than apterous individuals [65]. It may be possible that the red pea aphids are more active than the green aphids in terms of dispersal both by walking and flying away from UV-B radiation.

We then further investigated the effects of UV-B treatment on the activities of three protective enzymes in the pea aphid and revealed that the activity of all the assayed protective enzyme was affected in the UV-B treated aphids compared to those in untreated aphids (Figure 4). These enzymes (SOD, POD and CAT), which can effectively inhibit the active oxygen species from damaging and play an important role in maintaining normal functions of living organisms [66]. These three enzymes contribute to keeping cellular free radicals at a normal level, thus abating the damage of free radicals and raising the resistance of in living organisms to exogenous substances [67,68,69,70]. The effect of the UV-B treatment on the SOD activity increased with generation, but only after G_5_ generation was the SOD activity significantly increased by all UV-B treatments and reached a maximum level at 50 min treatment (Supplementary Figure S3A). The UV-B radiation first increased and then decreased the activities of POD in *A. pisum*, and these decreases were greater with increasing UV-B treatment time (Figure 4C,D), consistent with the finding on *Sitobion avenae*. [25]. The short-term UV-B treatments promoted the CAT activity of both red and green aphid morphs (Figure 4E,F), probably as a response to overcome UV-B-induced oxidative impacts. However, the CAT activity then declined with increasing generation. After the G_4_ generation, the CAT activity was lower than in the control under 50 min treatments (Supplementary Figure S3E,F). Interestingly, we found that the CAT activity was higher in the green aphids than in the red aphids, but at G_7_ generation, the activity of SOD and POD was higher in the red aphids than in the green aphids after the UV-B treatments (Figure 5), which may be why the red aphids are more adaptable to UV-B radiation and the fecundity is still lower in the red aphids than in the green aphids.

## 5. Conclusions

In this study, we demonstrated that UV-B radiation can lead to increase sugar contents in both red and green morphs of *A. pisum*, and confirmed the effects of UV-B radiation on the aphid physiology by means of regulating the protective enzyme activity. More interestingly, our study discovered the differences between the red and green aphids in response of UV-B radiations. Moreover, the sugar content, and protective enzymes (SOD and POD) activity of the red pea aphid are greater than those of the green pea aphid in long-term UV-B radiation, indicating the red aphid may be more adaptable to ultraviolet radiation.

## Figures and Tables

**Table 1:** Summary of analysis of two factors ANOVA results for effects of UV-B and generation interaction on the sugar contents and protective enzymes in *A. pisum*.

Measurement	Morph	Type III Sum of Squares	Df	Mean Square	*F*-Value	*p*-Value
Trehalose	Red	1.61	28	0.06	2.85	<0.001
	Green	0.80	28	0.03	1.48	0.089
Glycogen	Red	4.62	28	0.17	3.89	<0.001
	Green	2.66	28	0.10	4.88	<0.001
Protein	Red	489.78	28	17.49	12.09	<0.001
	Green	352.13	28	12.58	12.91	<0.001
SOD	Red	701.47	28	25.05	54.56	<0.001
	Green	300.34	28	10.73	31.71	<0.001
POD	Red	3.28	28	0.12	0.54	0.966
	Green	20.08	28	0.72	1.78	0.024
CAT	Red	36.92	28	1.32	4.90	<0.001
	Green	27.46	28	0.98	2.88	<0.001

## Data Availability

The datasets in this study are available from the corresponding author on reasonable request.

## Supplementary Materials

The following are available online at https://www.mdpi.com/article/10.3390/insects12121053/s1, Table S1: Average content (μg/mg) of total protein, Figure S1: Effects of UV-B radiation on trehalose content of *Acyrthosiphon pisum* for eight (G_0_–G_7_) generations, Figure S2: Effects of UV-B radiation on glycogen content of *Acyrthosiphon pisum* for eight (G_0_–G_7_) generations, Figure S3: Effects of UV-B radiation on the protect enzyme activity of Acyrthosiphon pisum at each of eight generations(G_0_–G_7_).
