# Supplementary material for: The Impact of Ultraviolet-B Radiation on the Sugar Contents and Protective Enzymes in Acyrthosiphon pisum
Source: Insects. 2021 Nov 25;12(12):1053. doi: 10.3390/insects12121053 (PMC8708437; doi:10.3390/insects12121053)

Supplementary Table S1. Average content (μg/mg) of total protein

Color morphs	Treatment time	Generation							
		G_0	G_1	G_2	G_3	G_4	G_5	G_6	G_7
Red	0(CK)	35.5±0.6Aab[1]	36.0±0.4Aab	36.1±0.3Ab	35.4±0.1Ad	35.6±0.6Ab	36.3±0.6Aa	35.9±0.8Aa	35.8±0.2Aa
	20	36.3±0.3DEa	37.8±0.6CDa	41.0±0.1Ba	46.3±0.1Aa	39.2±0.6BCa	35.4±0.5Ea	32.7±0.8Fb	31.5±0.2Fb
	30	33.1±0.5Bab	33.5±0.8Bbc	36.9±1.7Bb	41.6±0.3Ab	36.0±0.1Bb	34.7±0.6Ba	28.04±0.6Cc	27.52±0.8Cc
	40	32.8±1.3Bab	33.0±0.82Bbc	35.8±0.7ABb	38.6±0.6Ac	35.5±0.3ABb	33.5±1.1Ba	27.9±0.1Cc	25.0±0.5Cd
	50	31.6±1.5ABb	31.3±1.1ABc	30.6±0.6ABc	32.4±0.2Ae	27.9±1.1BCc	24.2±0.1CDb	24.0±0.1CDd	23.8±0.2Dd
Green	0(CK)	36.7±0.7Aa	36.8±0.5Ab	37.2±0.3Ab	36.2±0.2Abc	36.1±0.1Aab	36.3±0.4Aa	36.5±0.3Aa	36.4±0.6Aa
	20	36.7±0.3Ca	38.4±0.2Ca	40.8±0.1Ba	45.0±0.3Aa	38.7±0.9Ca	33.8±0.7Dab	31.9±0.2DEb	30.1±0.2Ebc
	30	34.1±0.1CDb	37.4±0.3Bab	40.3±0.5Aa	42.7±1.3Aa	35.7±0.3BCab	32.1±0.2DEbc	31.0±0.8Ebc	30.8±0.2Eb
	40	32.1±0.1BCc	33.2±0.2BCc	35.3±0.4ABb	37.7±0.4Ab	33.9±1.0BCbc	31.2±1.1CDbc	29.1±0.3Dc	29.3±1.4Dbc
	50	30.0±0.1BCd	29.1±0.1BCd	31.5±1.0ABc	33.1±0.7Ac	31.7±1.0ABc	30.5±0.7ABc	27.1±0.1Cd	27.1±.2Cc

[1] Effects of UV-B treatment and generation on the protein content of red and green aphids. Data are expressed as mean ± SE (n = 3). Different uppercase and lowercase letters indicate significant differences the effect of each UV-B radiation between generations (rows) and the effect of each generation between UV-B radiation (column), Respectively, ($p < 0.05$).

Supplementary Figure S1. **Effects of UV-B radiation on trehalose content of *Acyrthosiphon pisum* for eight (G_0-G_7) generations.** Different lowercase letters indicate significant differences between generations within the same UV-B radiation in red (A) and green (B) pea aphids. (*P* < *0.05*, Tukey-HSD test).

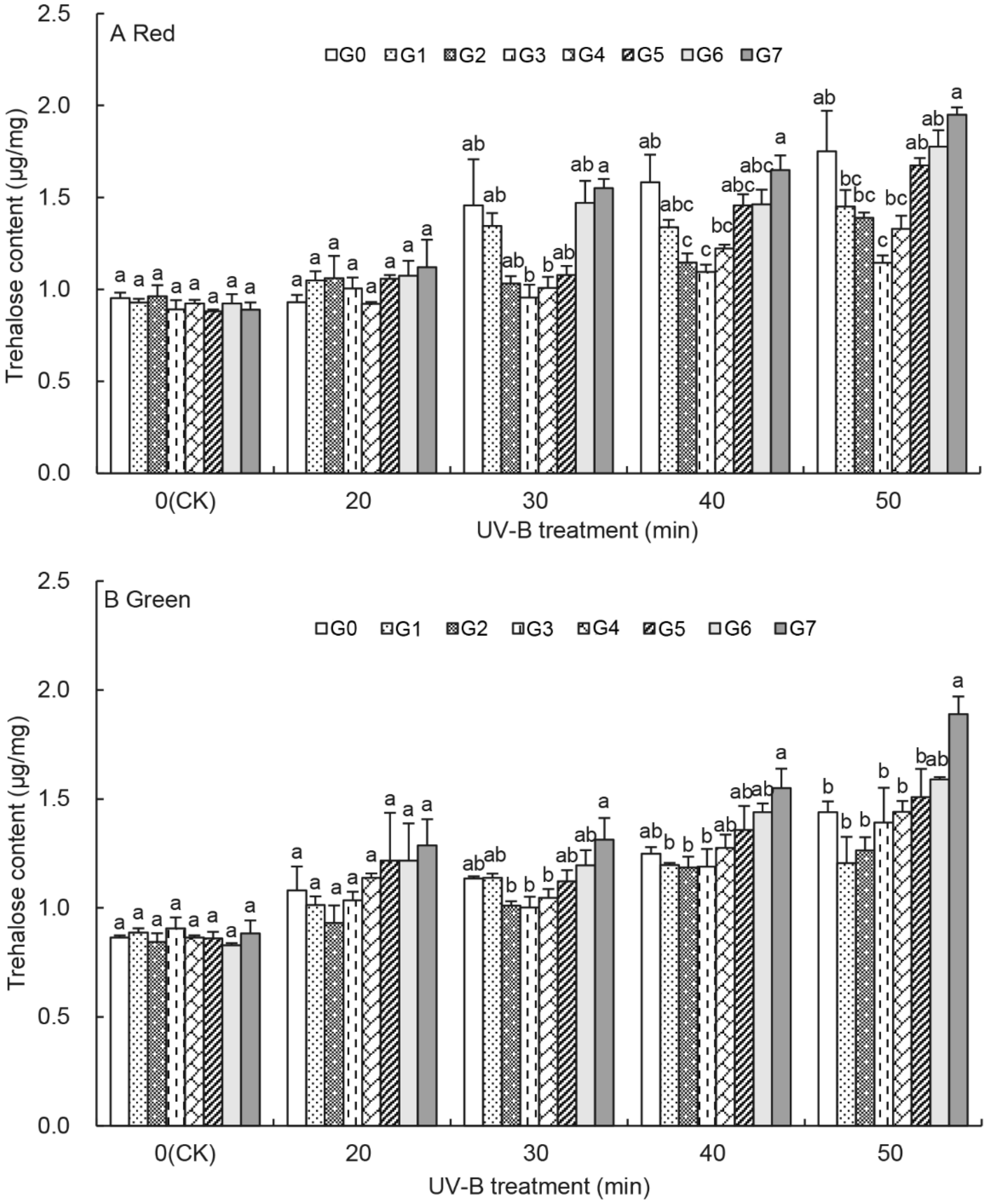

Supplementary Figure S2. **Effects of UV-B radiation on glycogen content of *Acyrthosiphon pisum* for eight (G_0-G_7) generations.** Different lowercase letters indicate significant differences between generations within the same UV-B radiation in red (A) and green (B) pea aphids. (P < *0.05*, Tukey-HSD test).

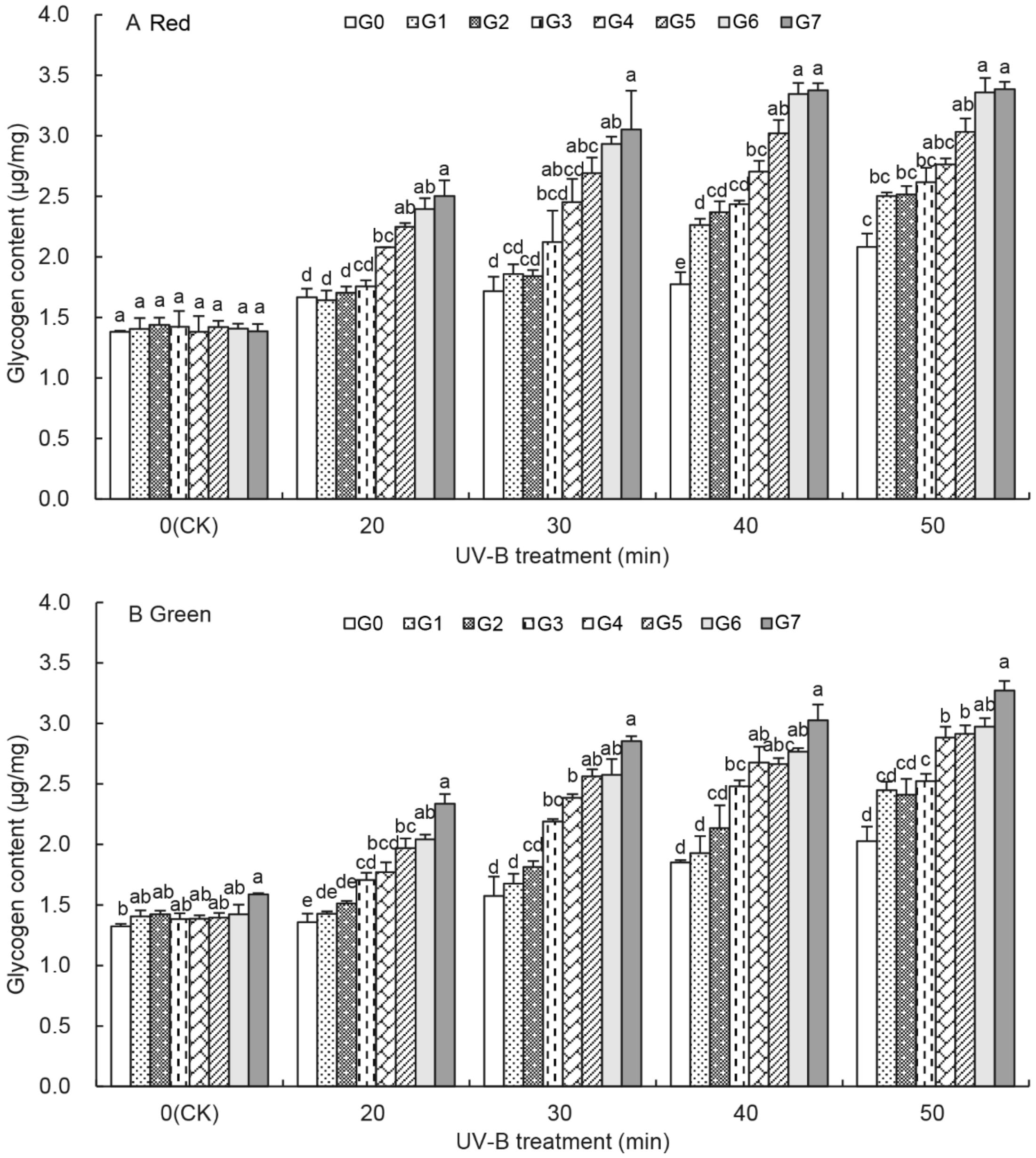

Supplementary Figure S3. Effects of UV-B radiation on the protect enzyme activity of *Acyrthosiphon pisum* at each of eight generations(G_0-G_7). A and B: Superoxide dismutase (SOD). C and D: Peroxidase (POD). E and F: Catalase (CAT). Different lowercase letters indicate significant differences between generations within the same UV-B radiation in red (A, C and E) and green (B, D and F) pea aphids. (*P* < *0.05*, Tukey-HSD test).

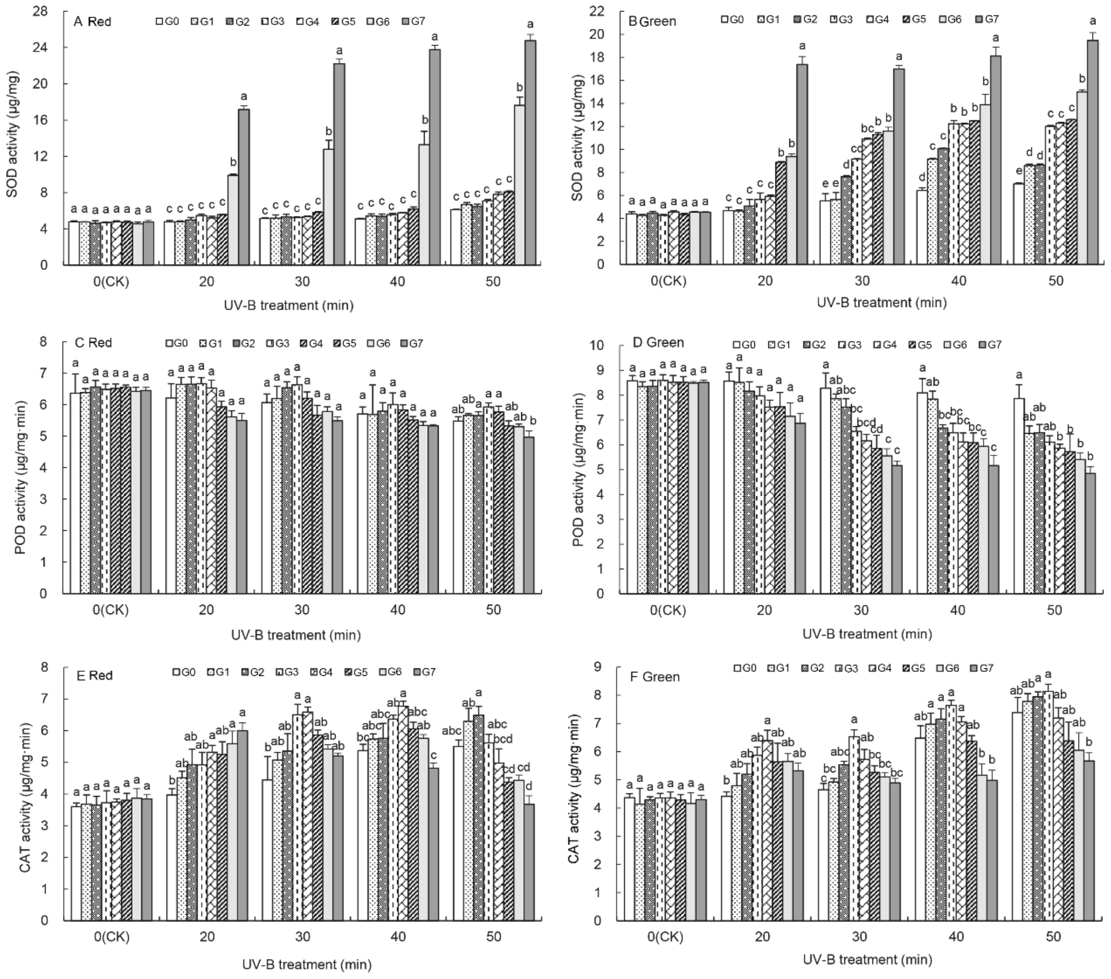

Supplement: Supplementary file 1 [file insects-12-01053-s001.zip › insects-1435120-SI.pdf]
